# Coupled Ionic-Electronic Equivalent Circuit to Describe Asymmetric Rise and Decay of Photovoltage Profile in Perovskite Solar Cells

**DOI:** 10.1038/s41598-019-48505-6

**Published:** 2019-08-19

**Authors:** Firouzeh Ebadi, Masoud Aryanpour, Raheleh Mohammadpour, Nima Taghavinia

**Affiliations:** 1 Institute for Nanoscience and Nanotechnology, Sharif University of Technology, Tehran, 14588 Iran; 2 Department of Mechanics, Sharif University of Technology, Tehran, 14588 Iran; 3 Department of Physics, Sharif University of Technology, Tehran, 14588 Iran

**Keywords:** Characterization and analytical techniques, Solar cells

## Abstract

In this research, we employed transient photo-voltage rise and decay measurements to investigate the origin of slow unsymmetrical rise and decay profiles in single and triple cation perovskite solar cells. Drastic changes in photo-voltage decay profile were observed upon insertion of Br^−^, Cs^+^ and FA^+^ ions into perovskite structures. In order to explain our observations, the activation energy for ionic defects was measured and an equivalent circuit model was proposed containing both electrical and ionic components. The electrical branch consists of a diode, the bulk capacitance and resistances for charge transport and recombination. In parallel we introduced an ionic branch describing the ionic response by a resistance for ionic charge transport and a capacitance describing ion accumulation at the interface to the charge transport layer. To reproduce the asymmetry of photo-voltage rise and decay, a diode with a parallel resistor is introduced leading to a belayed backflow of the accumulated ions. The results revealed that the activation energy of ionic defects became larger upon insertion of either halides or cations. There is larger amount of ionic defects in the case of MAPbI_3_ while the de-accumulation process of ions happens in much larger time scale in triple cation perovskite. The presence of ions at the interfaces results in band bending generating a potential barrier restraining electrons and holes from recombination; so the loss of built-in potential is delayed until de-accumulation of ionic double layer happens. Our model proposes that the loss of built-in potential depends on electrostatic potential drop, suggesting coupled electronic-ionic phenomenon in perovskite solar cells.

## Introduction

Organo-metal-halide perovskite solar cells are the subject of many ongoing research works in photovoltaics. Despite the fast growing efficiency of organo-metal perovskite solar cells, there are important questions around their operational principles^1–5^. A thorough understanding of the physical processes involved in the operation of perovskite solar cells is essential for achieving stable and high-performance devices. Ion migration is one of the most frequently stated problems with perovskite solar cells. It has received broad attention due to its significant impacts on the key operating parameters of the organo-metal perovskite solar cells^6–11^. In addition to the I-V hysteresis, ion migration is suspected as the origin of intrinsic instability of the fabricated devices^12^. Although extensive research has been carried out on ion migration in organo-metal perovskite materials, the experimental data are rather controversial, and a systematic understanding of how ion migration affects cell parameters is still lacking.

Here in this study we try to find out the reason for unsymmetrical photo-voltage rise and decay profile of perovskite solar cells, which is unconventional compared to previous types of photovoltaic cells such as dye sensitized solar cells. It has been seen that in contrast to what would be known in previous generations of solar cells, the photo-voltage decay does not show a simple exponential relaxation profile which is the sign of first order electron-hole recombination^13^. Bertoluzzi *et al*. suggested that open circuit voltage decay may involve cooperative relation kinetics and defined a “relation lifetime”^14^. Baumann *et al*. identified three distinct time regimes (μs, ms, s) during photovoltage decay and coupled electronic-ionic kinetics was proposed as an explanation^15^. Bisquert *et al*. provided a comprehensive description of perovskite solar cells physics and connected the slow processes with ionic charge layers near the selective contacts^16^. Moia *et al*. describe the perovskite-contact interfaces as transistors which couple ionic charge redistribution to energetic barriers controlling electronic injection and recombination^17^. Wang split open circuit voltage decay into two main parts and attributed the fast part to the depolarization of perovskite film and the slow part to interfacial charge recombination; however here we find that the slow and fast voltage loss are not independent and slow depletion results in change in photo-voltage decay profile^18^. Therefore, in this study we consider the unsymmetrical profile of both rise and decay curves of photo-voltage; additionally using various compositions including MAPbI_3_, MAPb(I,Br)_3_, CsFAMAPb(I,Br)_3_ we try to explain the extensive dissimilar behavior of photo-voltage rise and decay profile in perovskite solar cells. To achieve this, an equivalent circuit was proposed having both electronic and ionic components to simultaneously fit the photo-voltage rise and decay curvesin a precise way and interpret the underlying physical processes. Compared to previous equivalent circuits, we added a diode to the ionic branch that accounts for the different dynamics of photovoltage rise and decay. All experiments were carried out in various range of temperatures to distinguish the role of ionic and electronic components. Considering that some significant consequences including immobilization of ions at the interface, diffusion of ions through organic contacts^19^, and I-V hysteresis have been attributed to mobile vacancies^20^, investigating the impact of different halides and cations present in the structure on ion migration is of high importance.

## Results and Discussion

**Figure 1:**
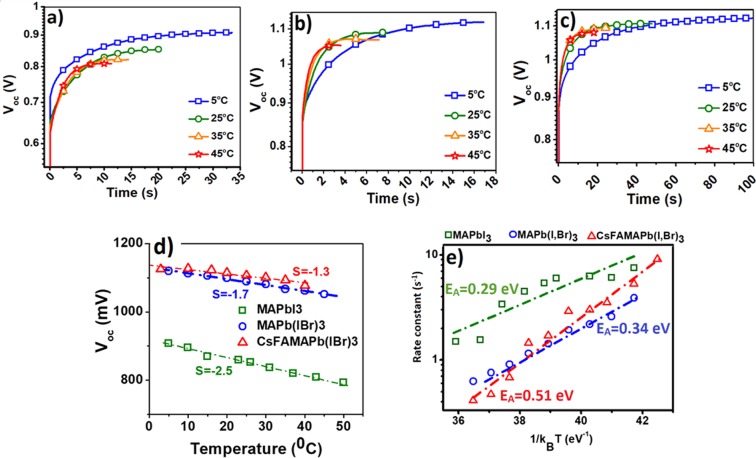
Open circuit voltage rise profile of solar cells based on (**a**) MAPbI_3_, (**b**) MAPb(I,Br)_3_, (**c**) CsFAMAPb(I,Br)_3_. (**d**) Reduction rate of steady state open circuit voltage by increasing the temperature for solar cells employed different absorbers. (**e**) Arrhenius plot and extracted activation energy for three different devices.

Temperature dependent photo-voltage rise characteristics of three types of perovskite solar cells, immediately after illumination, have been illustrated in the diagrams of Fig. 1a–c. One notes a rapid rise followed by a slow saturation of the photo-voltage to a final value. Both photo-voltage rise profile and final *V*_oc_ are dependent upon temperature and the perovskite composition. Decreasing the saturated photo-voltage via increasing the temperature is expected and can be an evidence of increased interfacial recombination, which appears to be more severe in the case of MAPbI_3_ device as shown in Fig. 1d. Studies by Chin *et al*. indicates that the field-effect electron and hole motilities gradually decrease by increasing the temperature as charge carriers are screened by mobile ions^21^; so the thermally ionized defects may be responsible for such a temperature dependent decrease in open circuit voltage (V_oc_). Based on Fig. 1d, the apparent decrease in the slope of curves for perovskite solar cells containing Br^−^, FA^+^, and Cs^+^ can be the evidence of an increase in defect activation energy; this can arise from stronger bonds of the crystal structure that make it difficult for ions to move across the layer^22^. Comparison of temperature dependent open circuit photo-voltage rises in some devices with different perovskite compositions predicts that devices with mixed halides and mixed cations show higher ion activation energy than MAPbI_3_. As presented in Fig. 1, V_oc_ rise profile of the cells shows two distinct fast and slow parts; the former caused by instantaneous built-in potential formation upon light incidence and the latter comes from progressive interfacial ion charging^23^. In order to investigate the ion migration activation energy, the slow part of the photo-voltage rise is fitted by a one-exponential growing function. As displayed in Fig. 1e, the activation energy can be extracted by plotting the logarithm of rate constant against 1/K_B_T^24^. The obtained value of activation energy is 0.29 eV for MAPbI_3_; by adding Br to the structure we have an increase in activation energy (0.34 eV); however, incorporation of FA^+^ and Cs^+^ in the perovskite structure leads to a much higher value for activation energy (0.51 eV). These values are in agreement with the results of most reports referred to activation energy for halide ion movement in perovskite film^24^. Most of calculation and experimental reports have shown the activation energy for halide ion migration in perovskite film within the range of 0.08–0.58 eV. The reported values for other existing ions are higher than 0.8 eV^25^. Considering that the obtained activation energy for these three types of solar cell with varied perovskite compositions are below 0.60 eV, it can be concluded that these values are related to halide ions. Consequently, the presence of the Br^−^, FA^+^, and Cs^+^ leads to increased activation energy. This is consistent with the recent reports, demonstrating that mixed organic cation perovskite resulted in stabilized crystal structure^4^. In the case of FA^+^, larger ionic size (radius = 0.279 nm) in comparison to MA^+^ (radius = 0.270 nm) and also the formation of hydrogen bonds with metal halide octahedral may contribute to stabilizing the lattice; however, strong electrostatic interaction of small size Cs^+^ (radius = 0.181 nm) with Br^−^ and I^−^ stabilizes the crystal structure^4^.

**Figure 2:**
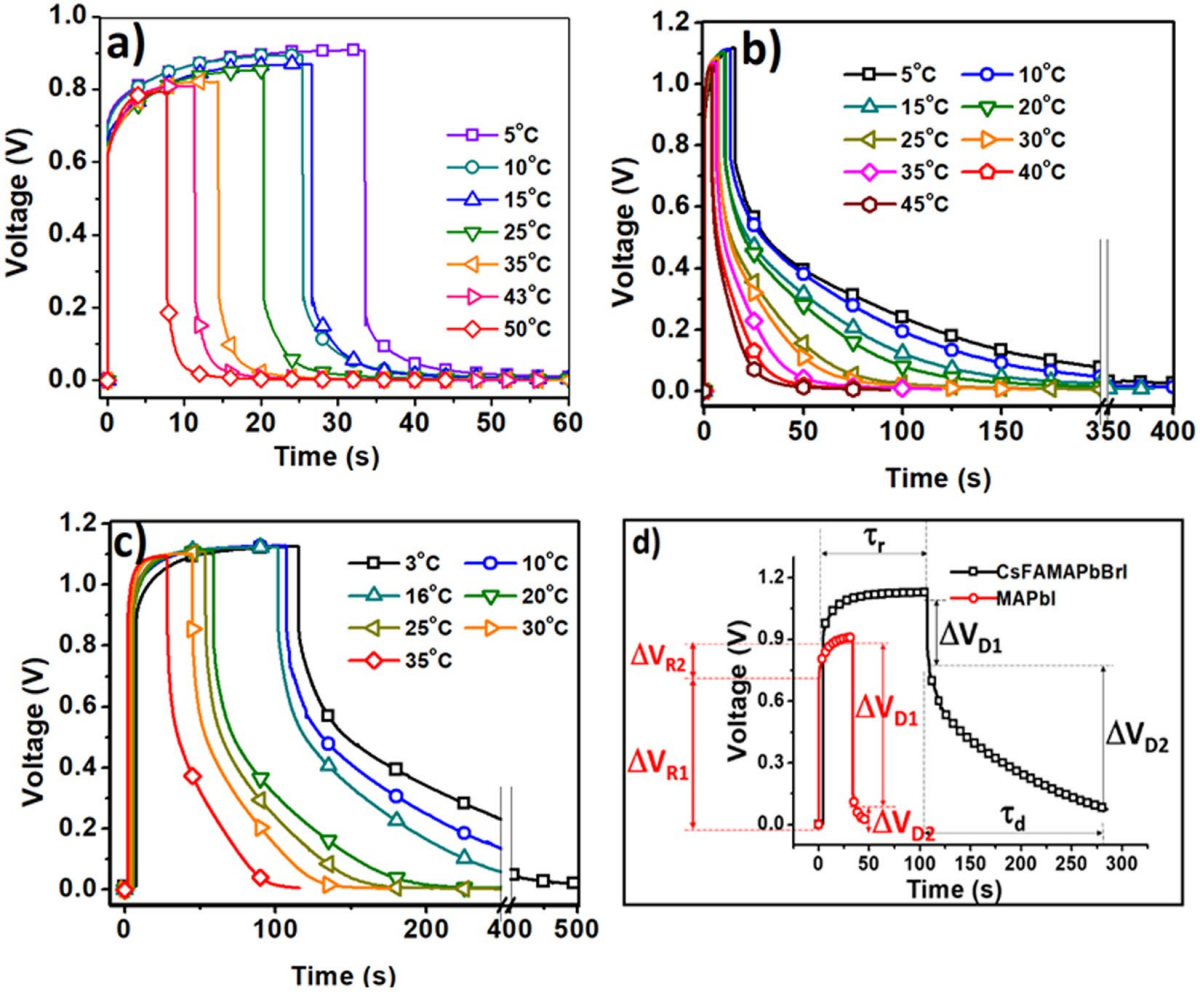
Photovoltage rise and decay profile of solar cells based on (**a**) MAPbI_3_, (**b**) MAPb(I,Br)_3_, (**c**) CsFAMAPb(I,Br)_3_. (**d**) Schematic of photovoltage rise and decay and the defined parameters; two stages with different behaviors are identified in rise and decay profiles.

To investigate the influence of mobile ions on cell performances, the time-domain photo-voltage rise and open-circuit voltage decay were recorded at various temperatures (Fig. 2a–c). Photo-voltage rise and decay profiles of the devices indicate several significant points: (a) at the first sight, it is obvious that the photo-voltage rise and decay profile does not have a symmetric behavior which is common for other types of solar cells such as dye sensitized solar cells. (b) photovoltage rise profile shows two distinct parts of fast and slow behaviors (Fig. 2d). All the extracted data were illustrated in Table S1. For all types of solar cells the initial fast rise leads to gain a main portion of open circuit voltage (ΔV_R1_) (~650 mV for MAPbI_3_, ~860 mV for MAPb(I,Br)_3_ and ~880 mV for CsFAMAPb(I,Br)_3_) less than 50 ms after turning on the LED, followed by a small potential rise (ΔV_R2_~200 mV) with slow rise profile in multi-seconds (τ_r_). (c) V_oc_ decay also displays fast and slow decay profile. In case of the device with MAPbI_3_ the fast drop leads to large loss of V_oc_ (ΔV_D1_~ between 550 mV to 720 mV) less than 50 ms after turning off the LED followed by a slow drop (ΔV_D2_ ~ 200 mV) after multi-seconds. This behavior for MAPbI_3_ composition has been seen in the previous reports which attributed these two parts to built-in and electrostatic potentials, respectively. However adding Br^−^, Cs^+^ and FA^+^ cations to perovskite composition causes a large difference in decay profile. As seen in the diagrams of Fig. 2b,c, the fast decay (ΔV_D1_) (less than 50 ms) drops only in the range of 200 mV to 300 mV, subsequent to slow part of decay (ΔV_D2_) after passing a few tens of seconds (τ_d_). Such a large difference in the value of fast decay for different compositions of the perovskite can reflect significant physical phenomena behind this feature which will be discussed later. (d) By increasing the temperature, the slow and fast time constants become smaller, that is reasonable since the migration of ions towards interfaces and vice versa is thermally activated and is faster for higher temperatures.

**Figure 3:**
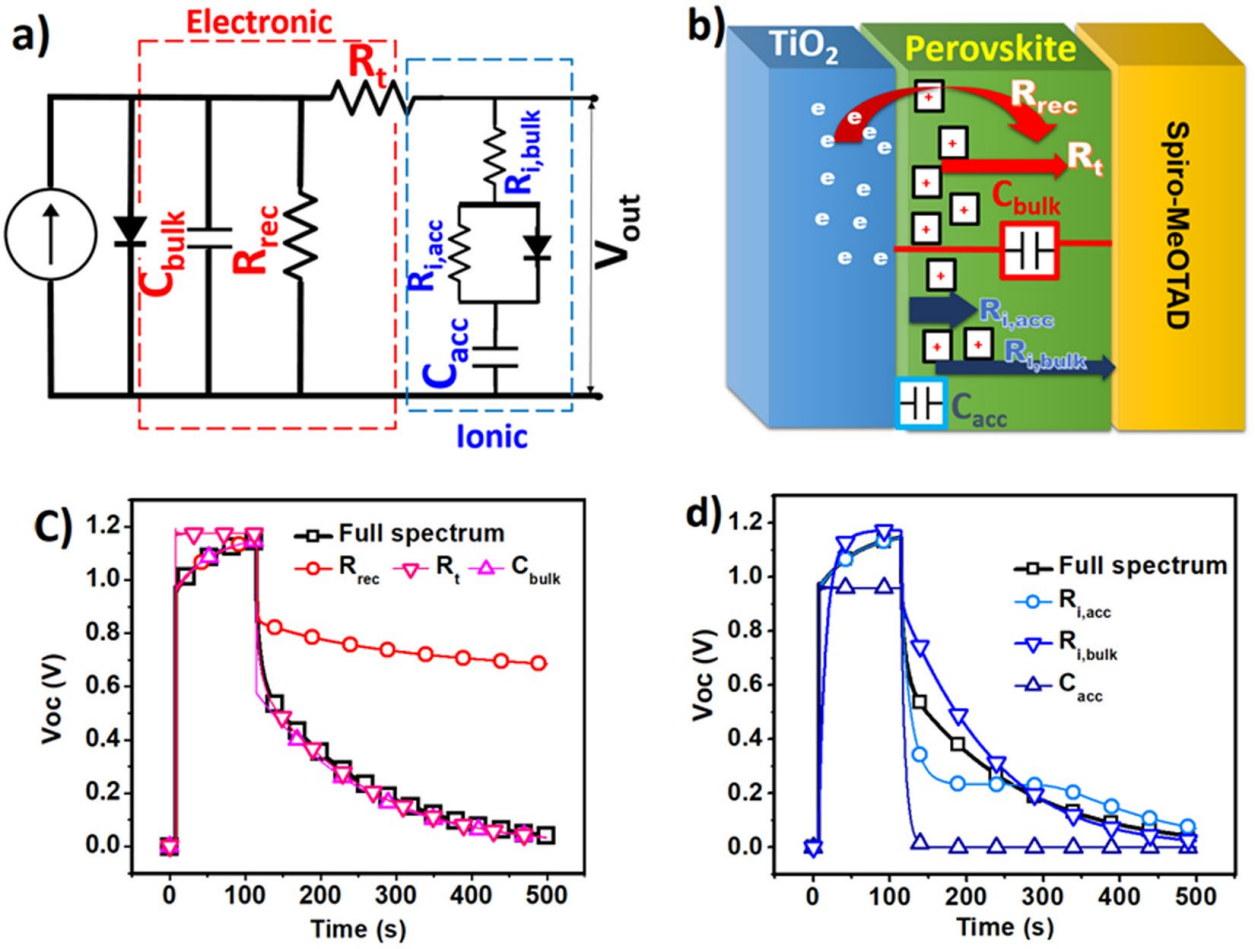
(**a**) Equivalent circuit for fitting photo-voltage rise and decay plots. MAPbI_3_, (**b**) schematic of layers of perovskite based solar cells and defined parameters. Effect of removing (**c**) electronic and (**d**) ionic elements on the fitted data related to V_oc_ decay and rise curves.

To understand and analyze the physical processes quantitatively, an equivalent circuit to model the photovoltage rise and decay has been proposed which accounts both electronic and ionic processes. We tried to simultaneously fit the data of unsymmetrical rise and decay profile by employing this circuit. As illustrated in Fig. 3a, two parallel parts have been considered in the proposed circuit. One for ionic process and the other represents the equivalent elements relating to common electronic processes. The schematics for the related elements has been demonstrated in Fig. 3b. In the electronic branch which is correlated to the electronic contribution of photo-generated carriers, R_rec_ refers to back transfer resistance for recombination of electrons in TiO_2_, C_bulk_ represents the bulk capacitance of perovskite and all types of electrical charge storage in perovskite solar cell including geometrical, chemical and electronic contact capacitances have been considered in this element. R_t_ corresponds to carrier transport resistance. In the ionic branch, R_i,bulk_represents the ionic transport resistance of perovskite material and C_acc_ is the capacitance formed as a result of ion accumulation at the interface of electron and hole transport material and perovskite material. R_i,acc_refers to the resistivity against the accumulated ions at the interface to return back into the bulk of film. A diode is used in parallel with R_i,acc_ which represents unsymmetrical charging and discharging of the interface, which leads to easy trapping of ionic charges at the interface and more difficult de-trapping in the bulk. This description is consistent with recent KPFM (Kelvin Probe Force Microscopy) study revealing different times for charging and discharging of interface^26^. All photo-voltage rise and decay profiles were fitted through the equivalent circuit of Fig. 3a and fitted curves for various temperatures have been illustrated in the Figs S1–S3 in the supplementary information. In order to verify how each individual model element affects the rise-decay profiles, the modeling was performed separately in the absence of the same element, as illustrated in Fig. 3c,d. The graphs show that removing R_rec_ and C_bulk_ elements predominantly influences the V_oc_-decay profile, while omitting R_t_ mainly affects the photo-voltage rise character. The slow part of decay profile becomes much slower upon removing R_rec_ and as it is expected after discharge of capacitances there is no way for photo-carriers to recombine. Conversely, taking out R_t_ element leads to a very fast rise of photo-voltage and so the slow rise profiledisappears. When ionic elements are removed, Fig. 3d, deleting R_i,bulk_ and R_i,acc_causes the change in both rise and fall profiles to some extent. But the main changes happen upon removing C_acc_. Removing C_acc_ results in very fast rise and decay behavior as it happens commonly in typical solar cells and both slow and fast rise and decay profile disappear. Additionally, the maximum achieved V_oc_ decreases as it is expected, as internal electrostatic potential is removed upon deleting charge accumulation in the interfaces.

**Figure 4:**
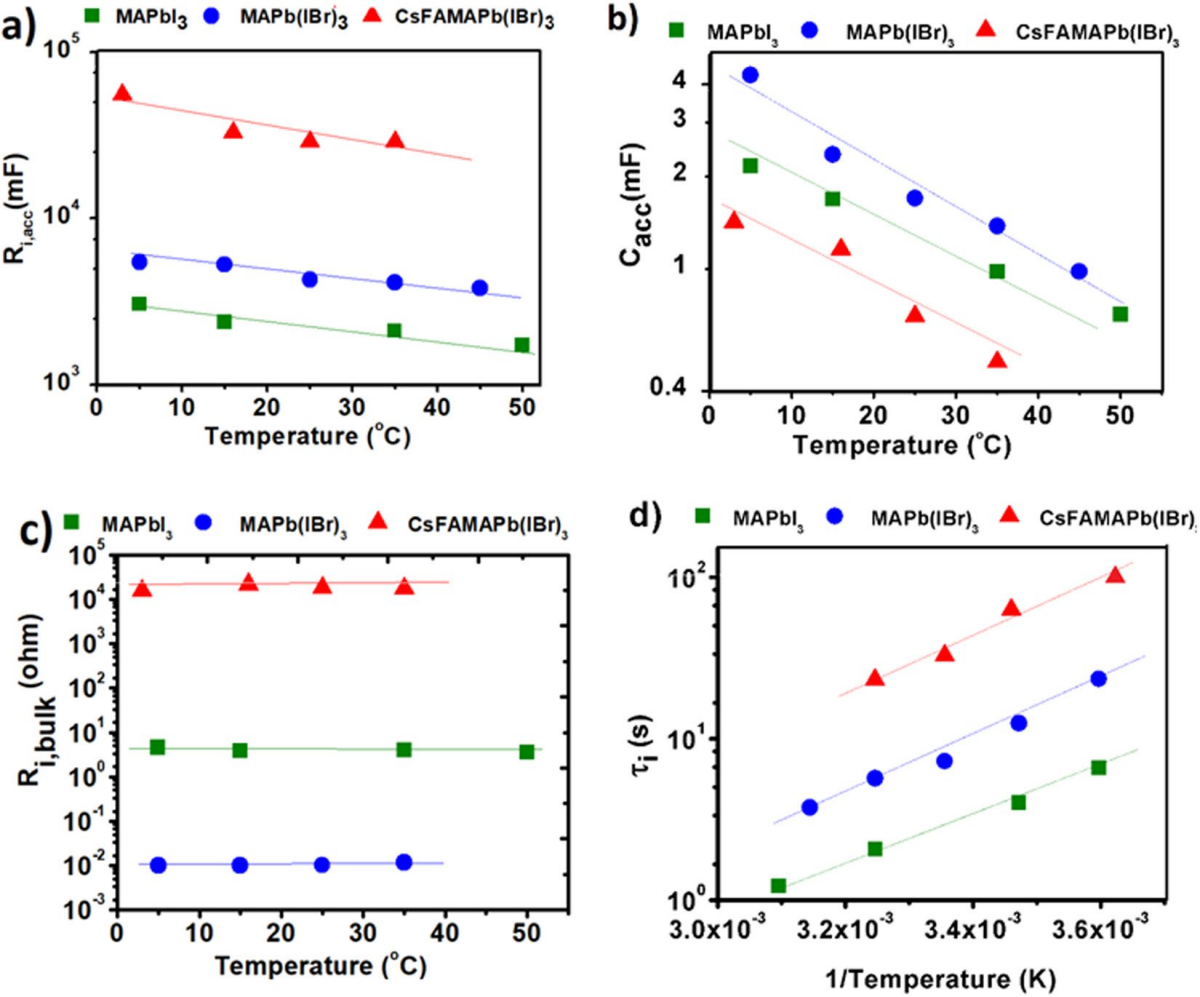
Dependence of (**a**) ionic accumulation resistance, R_i,acc_, (**b**) accumulation capacitance, C_app_, (**c**) ionic transport resistance, R_i,bulk_ and (**d**) ionic characteristic time, τ_i_ as a function of temperature.

Figure 4 represents the fitting values for ionic components employing the proposed equivalent circuit. The change in R_i,acc_value which is the resistivity against the accumulated ions at the interface of TiO_2_/perovskite film to return back into the bulk (after turning the light off) has been illustrated in Fig. 4a. It is obvious that the amount of R_i,acc_ is intensely dependent to perovskite composition as it is one order of magnitude higher in CsFAMA(I,Br)_3_ in comparison to MAPbI_3_. This indicates that as the mobile ion defects migrate to the interface due to photo-carrier generation and induction of internal electric field, the accumulated ions in CsFAMA(I,Br)_3_ move back much slower into the bulk of perovskite after turning the light off. There is a slight decrease in R_i,acc_ values upon increasing temperature which can be accepted as a result of gaining more thermal activation energy to de-trap from the ionic accumulation layer. Conversely, C_i,acc_ which relates to accumulated ions in the interface of TiO_2_ and perovskite film decreases upon inserting FA^+^ and Cs^+^ as can be seen in the diagrams of Fig. 4b; this may result from lower density of mobile ion defect due to higher activation energy. C_i,acc_ decreases by increasing the temperature and is compatible to the Gouy-Chapman model. As it can be seen in the diagram of Fig. 4c, the bulk ionic resistance, R_bulk_, depends on the perovskite composition and it is much larger for perovskites containing Br^−^, FA^+^, and Cs^+^; One can conclude that increase in the activation energy (Fig. 1e) makes it difficult for ions to be mobile through the film. Based on these results, upon inserting Cs^+^ and FA^+^ into the perovskite structure, lower density mobile ion defects are created in the film (after turning the light on) resulting in lower values of ionic capacitance, however after turning the light off the resistance of ions of accumulation layer to returning back in triple cation perovskite structures gets much higher in comparison to MAPbI_3_. Throughout our descriptions, type of migrating ion species has not been specified and it would be the subject of upcoming research.

To evaluate the decay process, the time constant of ionic branch was calculated based on the analysis of equivalent circuit, τ_i_ = C_i,app_ * [R_i,bulk_ + R_i,acc_ * rD_1_/(rD_1_ + R_i, bulk_)], where r and D_1_ are the diode parameters. The calculated value of ionic characteristic times as a function of temperature are shown in the Fig. 4d. It is obvious that upon insertion of Br^−^, FA^+^, and Cs^+^ ions the characteristic time for ionic branch increases which shows itself in much slower voltage decay profile. The thermally activated ionic process shows itself in temperature-dependent ionic characteristic time (Fig. 4d).

**Figure 5:**
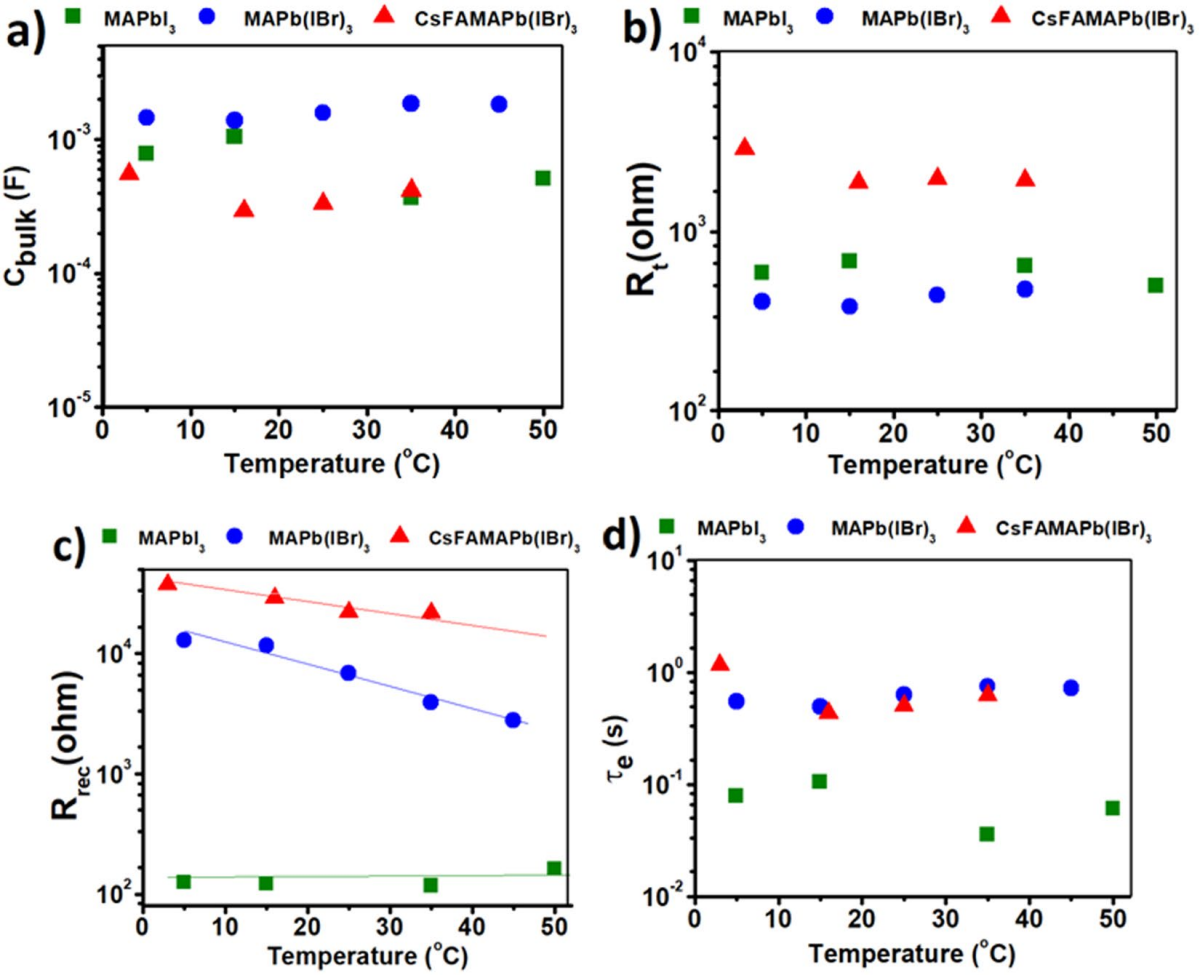
Dependence of (**a**) photo-induced bulk capacitance, C_bulk_, (**b**) transport resistance, R_t_, (**c**) recombination resistance, R_rec_ and (**d**) electronic characteristic time, τ_e_ as a function of temperature.

The values of electronic components have been fitted and their behavior was illustrated in Fig. 5. As expected there is no temperature dependent behavior evident in the values of C_bulk_ and R_t_ as indicated in the diagrams of Fig. 5a,b. However the values of back recombination resistivity, R_rec_, show logarithmic temperature dependence and also their values are more than two orders of magnitude higher in the case of CsFAMA(I,Br)_3_ (~30000 ohm) in comparison to MAPbI_3_ (100 ohm). Since the calculation proves that upon inserting Br^−^, FA^+^, and Cs^+^, a not so large electronic energy level shift happens, this amount of difference of back recombination resistivity cannot be attributed only to electronic-based effects. There should be some relationship between very large values of R_rec_ (Fig. 5c) and R_i,acc_ (Fig. 4a) which we discuss later. To investigate the electronic decay process, the time constant of electronic branch was calculated based on the analysis of circuit, τ_e_ = [C_bulk_/(1/R_rec_ + 1/rD_e_ + 1/R_t_)], where r and D_e_ are diode parameters in the electronic branch. The calculated values for electronic characteristic time as a function of temperature are shown in the Fig. 5d. No temperature dependent behavior is evident and the order of magnitude for electronic time constant becomes ten times larger upon inserting additive cations into perovskite structure.

**Figure 6:**
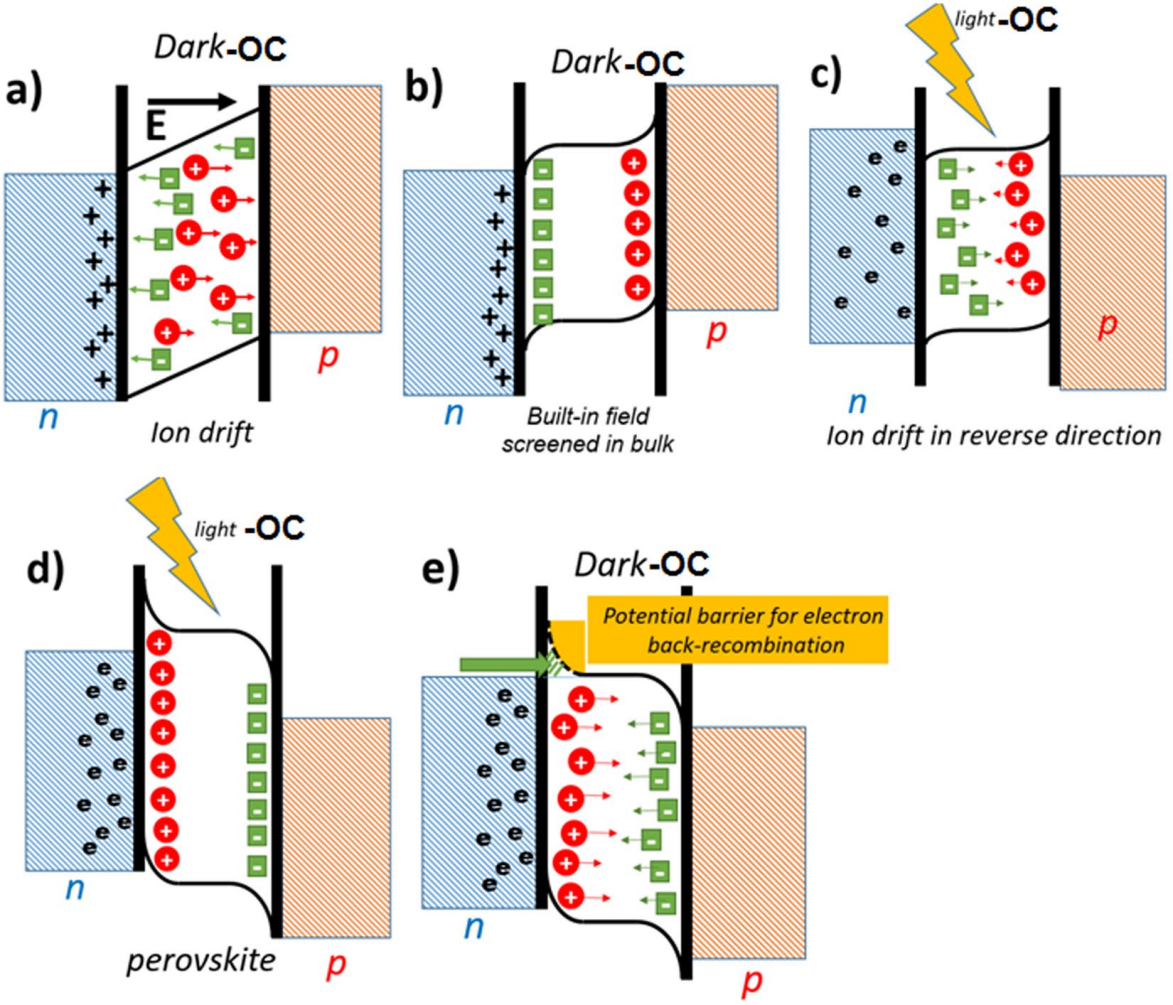
The energy diagram in perovskite absorber layer (**a**) Just after contact in dark, (**b**) in equilibrium in dark, (**c**) Just after light illumination, (**d**) in equilibrium under light, (**e**) after light soaking in dark.

Based on our experimental results and the values of electronic and ionic components extracted from our proposed equivalent circuit one can conclude that there should be some coupled electronic and ionic processes in perovskite solar cells which change the photo-voltage rise and decay profile through the change in perovskite composition. The effect associated with ion migration in MAPbI_3_ perovskite and redistribution of electric field in active layer has been described recently by Bisquert *et al*. Here we try to illustrate this phenomenon based on the values related to electronic and ionic elements. Based on our result the above mentioned process may happen as we measure the open-circuit transient profile upon turning on and off the light (Fig. 6). Through all description we present, the type and charge of ionic defects are not related to any specific ion and we discuss it in general. At equilibrium, the Fermi level of the perovskite, electron transport layer (ETL) and hole transport layer (HTL) are the same, generating an internal electric field (Fig. 6a) inside the perovskite layer. Cations and/or anions drift along the electric field to shield the field resulting in accumulation of anions at the interface of ETL and perovskite film while cations accumulate at the interface of HTL and perovskite material (Fig. 6b). Upon incidence of light, TiO_2_ becomes the reservoir of electrons and in the other side SPIRO is full of holes, so there is a change in the direction of internal electric field in perovskite layer; in this condition anions should migrate to HTL interface and cations move back towards ETL until the internal electric field is shielded once more (Fig. 6c). In quasi-stable situation in the open circuit condition under light, an accumulation of cations and anions will happen at the interface of perovskite with ETL and HTL respectively (Fig. 6d). In such condition the sum of built-in potential and electrostatic potential yields the achieved photo-voltage of solar cell. When the light is turned off anions and cations should de-accumulate respectively from HTL and ETL interfaces (Fig. 6e). There are two considerable points: (a) a potential reason for unsymmetrical behavior of photovoltage rise and decay can be the accumulation of ions with different signs in the beginning of the process of turning on and off the light. It means that in the equilibrium condition in dark, negative ions accumulate near ETL and positive ions accumulate close to HTL; while under semi-stable condition in dark, the reverse phenomenon happens. Since the trap-activation energy for cations and anions is likely to be different for depletion from the interfaces so we can expect for unsymmetrical photo-voltage rise and decay profiles. (b) As depicted in the schematics of Fig. 6e, when the photo-voltage is stabilized there is an accumulation of ions at the interfaces which results in formation of ionic double layer and energy band bending. As illustrated in decay profile of V_oc_, after turning the light off in the first step the electrons in TiO_2_ recombine with holes in perovskite layer, a behavior which shows itself in fast voltage decay profile (less than 50 ms), however the decay of remaining photovoltage is dependent to depletion of internal ionic double layer, since it forms a potential barrier restraining the electrons and holes from moving back to perovskite material and recombination. In case of inserting Br^−^, Cs^+^ and FA^+^ into perovskite structure, since the amount of R_i,acc_becomes much larger, so it takes much longer time for ions to deplete the ionic double layer. As a consequence, the back recombination of remained electrons and holes is up to the discharge of accumulation in double layer. So larger values of recombination resistivity have been achieved for perovskite solar cells employing MAPb(I,Br)_3_ and CsFAMAPb(I,Br)_3_. These achieved results indicate that the ionic-based capacitance is not the only reason for slow response of perovskite solar cell but the coupled electronic and ionic components also influence this behavior.

Based on our results the photo-voltage rise and decay profiles are affected extensively by the type of perovskite composition including MAPbI_3_, MAPb(I,Br)_3_, CsFAMAPb(I,Br)_3_. In fact, all types of perovskite solar cells display unsymmetrical rise and decay profiles, containing both fast and slow rise/decay. In the case of MAPbI_3_ photo-voltage substantially decays very fast (in initial 50 ms after turning the light off) followed by slow decay; however upon adding halides and cations to perovskite, the major part of photo-voltage is lost during slow decay profile. Based on our proposed equivalent circuit, the activation energy for defect ion formation is lower in MAPbI_3_ resulting in formation of larger ionic double layer capacitance, however the resistivity of ions to depletion of layer is lower in comparison to triple cation perovskite solar cell; this results in very fast voltage loss. Conversely, adding cations and halides results in reduced values of ionic defects and lower values of ionic double layer capacitance. However, the resistivity for depletion of ionic double layer is two orders of magnitude higher, resulting in much larger recombination resistivity. The accumulation of ion defects with different signs in dark and under light at the interface of perovskite and HTL and ETL leads to drastic unsymmetrical rise and decay profile of perovskite solar cells.

## Methods

### Experimental methods

#### Cell fabrication

Fluorine-doped tin oxide (FTO) glass sheet was etched with zinc powder and diluted HCl. Then the etched substrate was ultrasonically cleaned with detergent, deionized water, acetone and ethanol respectively. The compact TiO2 was coated by spin coating from a precursor solution of titanium containing 0.2 M titanium isopropoxide (TTIP, 97%, Merck) and 0.2 M HCl at 2000 rpm for 30 s, followed by annealing at 500 °C for 30 min. A 250 nm mesoporous TiO2 layer was deposited by spin-coating using 20 nm particle size paste (IRASOL PST-20T) diluted in ethanol. Then the substrate was dried at 100 °C for 10 min and sintered at 500 °C for 30. After cooling down to 150 °C, the substrates were immediately transferred to a nitrogen atmosphere glove box for depositing the perovskite films.

MAI, FAI were purchased from Dyesol; the lead compound from Borun New Material Technology Co; CsI from abcr GmbH. To prepare mixed-halides and cations perovskite precursor solution, we have followed a similar approach using the mixed “Cs0.05 (MA0.17FA0.83)0.95Pb(I0.83Br0.17)3” formulation that has been used by Michael Saliba. *et al*.^10^. The mixed perovskite precursor solutions was spin coated in a two steps program at 1000 and 4000 rpm for 10 and 20 s respectively. During the second step, 200 μL of chlorobenzene was poured on the spinning substrate after 15 second from the starting point of the second step. Then the substrates were annealed at 100 °C for 1 h. The mono cation perovskite solution (MAPbI3) prepared by dissolving 600 mg PbI2 and 183 mg MAI in 1 mL DMSO (MercK). Also, the solution of MAPb(IBr)3 perovskite prepared by substitution of 0.17 PbBr2 instead of PbI2. The mono cation perovskite films was spin coated in a two steps program at 1000 and 6000 rpm for 10 and 30 s respectively using 100 μL Chlorobenzene as the anti-solvent.

The hole transport layer was deposited by spin coating at 5000 rpm for 30 s, using the solution prepared by dissolving 72.3 mg Spiro-OMeTAD (99.5%, Borun New Material Technology Co), 28.8 μL 4-tert-butylpyridine, and 17.5 μL of LiTFSI solution (520 mg/mL LiTFSI (98%, Merck) in acetonitrile) in 1 mL chlorobenzene. As the last layer, Gold top electrode was thermally deposited under high vacuum. All device fabrication and characterization proccesses were carried out under ambient conditions with humidity around 20%.

#### Perovskite solar cell characterization

The performance of the devices was measured using a solar simulator (IRASOL, SIM-1000) at 100 mW cm^−2^ illumination AM 1.5G at a scan rate of 50 mV s^−1^. The open circuit voltage rise and decay measurement was done by excitation of cell employing 10 W green LED and the profile was measured by potentiostat/galvanostat (MetrohmAutolab).

### Computational methods

At each test temperature, the parameters of the equivalent circuit are optimized for the Voc-decay response of the call. The optimization procedure is as follows. The model of the circuit is translated automatically into an input file for the circuit solver (Ngspice) which includes the definition of all the passive and active elements, their circuit connections, well as the current source as a function of time. Ngspice, is a powerful circuit solver and quite capable of simulating complex circuits for both linear and nonlinear analyses (transient in our case). Ngspice also accepts user-defined elements and power sources, for example, a dc-pulse current source, and a custom diode, both needed in the proposed equivalent circuit.

The circuit parameters are optimized by minimizing the cost function, which is defined as the sum of absolute differences between the values of the output voltage and the corresponding measured response of the cell, as given by Equation (1):

1 $$error=\sum _{t}\,|V{(t)}_{experiment}-{\rm{V}}{(t)}_{simulated}|.$$

The sampling of simulation time is performed for the duration of the sensing before the beginning of the rise, and continued to the end, where the measured output is reported. The optimization loop, implemented in MATLAB environment to benefit from its powerful optimization toolbox functions, is then executed. The optimization proceeds to minimize the cost function by varying the circuit parameters until achieving a good match between the simulated and the measured voltage response.

## Supplementary information

SI
