# Supplementary material for: Coupled Ionic-Electronic Equivalent Circuit to Describe Asymmetric Rise and Decay of Photovoltage Profile in Perovskite Solar Cells
Source: Sci Rep. 2019 Aug 19;9:11962. doi: 10.1038/s41598-019-48505-6 (PMC6700164; doi:10.1038/s41598-019-48505-6)
Supplement: Supplementary file 1 — SI [file 41598_2019_48505_MOESM1_ESM.docx]

Coupled Ionic-Electronic Equivalent Circuit to Describe Asymmetric Rise and Decay of Photovoltage Profile in Perovskite Solar Cells

Firouzeh Ebadi^a^, MasoudAryanpour^a,b^, Raheleh Mohammadpour^*, a^, Nima Taghavinia^*, a,c^

^a^ Institute for Nanoscience and Nanotechnology, Sharif University of Technology, Tehran 14588, Iran

^b^ Department of Mechanics, Sharif University of Technology, Tehran 14588, Iran

^c^ Department of Physics, Sharif University of Technology, Tehran 14588, Iran

**Table S1.**Photovoltage rise and decay parameters related to solar cells with different absorber materials. Information are extracted from the curves of Figure 2.

| ***Absorber*** | *Temperature (K)* | ***photovoltage rise*** | ***(ΔV_R1_)****Initial Fast Voltage rise after 50 ms (mV)* | ***(τ_r_)*** *Time interval for full rise (S)* | ***(ΔV_R2_)*** *Slow oltage rise (mV)* | ***Photovoltagedecay*** | ***(ΔV_D1_)****Initial Fast Voltage decay after 50 ms (mV)* | ***(τ_d_)*** *Time interval for full decay (S)* | ***(ΔV_D2_)****Slow voltage decay (mV)* |
| --- | --- | --- | --- | --- | --- | --- | --- | --- | --- |
| ***MAPbI*** | *278* |  | *709* | *30* | *199* |  | *722* | *16* | *196* |
|  | *283* |  | *698* | *24.5* | *197* |  | *656* | *13.1* | *214* |
|  | *288* |  | *664* | *23.9* | *206* |  | *652* | *12.1* | *193* |
|  | *298* |  | *655* | *19.3* | *198* |  | *630* | *8* | *197* |
|  | *308* |  | *637* | *14.1* | *184* |  | *593* | *5.4* | *201* |
|  | *316* |  | *620* | *8.5* | *189* |  | *586* | *6.8* | *195* |
|  | *323* |  | *617* | *6.5* | *176* |  | *563* | *4.2* | *202* |
|  | | | | | | | | | |
| ***MAPbBrI*** | *278* | ***photovoltage rise*** | *865* | *15* | *254* | ***Photovoltagedecay*** | *369* | *390* | *726* |
|  | *283* |  | *878* | *10.8* | *234* |  | *346* | *264* | *741* |
|  | *288* |  | *867* | *9.7* | *239* |  | *345* | *184* | *736* |
|  | *293* |  | *859* | *8.8* | *242* |  | *336* | *144* | *731* |
|  | *298* |  | *854* | *6.6* | *235* |  | *340* | *91* | *723* |
|  | *303* |  | *863* | *4.4* | *217* |  | *333* | *76* | *720* |
|  | *308* |  | *862* | *4* | *208* |  | *331* | *53* | *712* |
|  | *313* |  | *852* | *3.5* | *210* |  | *330* | *40* | *705* |
|  | *318* |  | *869* | *2.6* | *184* |  | *332* | *30* | *694* |
|  | | | | | | | | | |
| ***CsFAMAPbBrI*** | *276* | ***photovoltage rise*** | *870* | *103* | *255* | ***Photovoltagedecay*** | *272* | *353* | *829* |
|  | *283* |  | *885* | *94* | *242* |  | *266* | *250* | *837* |
|  | *289* |  | *887* | *78* | *235* |  | *247* | *200* | *850* |
|  | *293* |  | *880* | *50* | *220* |  | *277* | *134* | *798* |
|  | *298* |  | *885* | *42* | *222* |  | *262* | *109* | *819* |
|  | *303* |  | *889* | *32* | *212* |  | *247* | *92* | *823* |
|  | *308* |  | *869* | *24* | *224* |  | *221* | *68* | *845* |


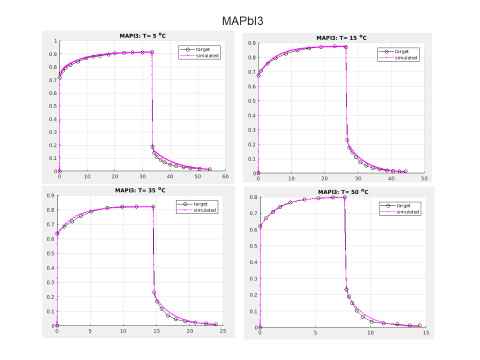


Figures S1: Fitted curves for photo-voltage rise and decay profiles of MAPbI3 were fitted through the equivalent circuit of Figure 3a


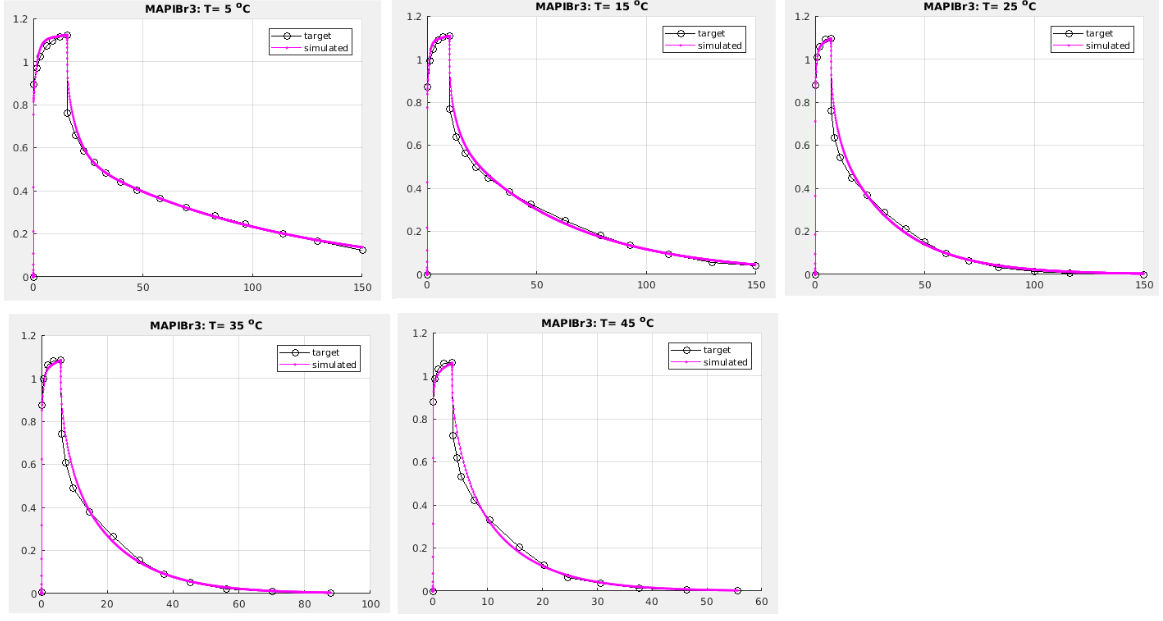


**Figures S2: Fitted curves for photo-voltage rise and decay profiles of MAPb(I,Br)_3_ were fitted through the equivalent circuit of Figure 3a**


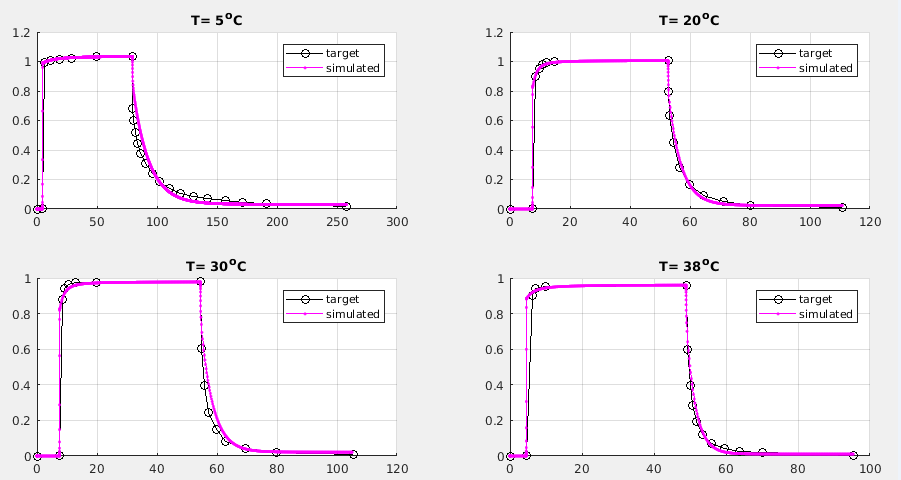


**Figures S2: Fitted curves for photo-voltage rise and decay profiles of CsFAMAPb(I,Br)_3_ were fitted through the equivalent circuit of Figure 3a**
